# Keratoameloblastoma of the jaws and review of international literature of 38 cases

**DOI:** 10.4317/jced.62973

**Published:** 2025-08-01

**Authors:** Rafael Netto, Maria Elisa Rangel Janini, Wladimir Cortezzi, Ludimila Lemes Moura, Silas Antonio Juvencio de Freitas-Filho

**Affiliations:** 1 Department of Oral Diagnosis and Pathology, School of Dentistry, Federal University of Rio de Janeiro (UFRJ), Rio de Janeiro, RJ, Brazil; 2 Head, Oral and Maxillofacial Surgery Service, Hospital Federal dos Servidores do Estado do Rio de Janeiro, Rio de Janeiro, RJ, Brazil; 3 Pontifical Catholic University of Minas Gerais (PUC Minas), Poços de Caldas, MG, Brazil; 4 University Center of the Associated Colleges of Education (UNIFAE), São João da Boa Vista, SP, Brazil; 5 Department of Surgery, Stomatology, Pathology and Radiology, Bauru School of Dentistry, University of São Paulo, Bauru, SP, Brazil

## Abstract

The term keratoameloblastoma has been used to describe a histologically heterogeneous group of ameloblastoma variants that share the formation of keratin by the ameloblastomatous epithelium. To date, thirty-eight cases of keratoameloblastoma have been previously reported in the literature, nine of which exhibited a papilliferous component. Here we report a new case of a recurrent tumor that falls within the keratoameloblastoma spectrum. It presented as an expansile, solid lesion with internal calcification in the right infratemporal fossa six years after ipsilateral hemimandibulectomy in a 46-year-old white female. Histological evaluation revealed islands of columnar cells resembling ameloblasts surrounding a central area with stellate reticulum-like cells, some of them completely filled with keratin. In addition, areas showed basal ranging from columnar to cuboidal with hyperchromatic nuclei. The clinical, histopathologic, and radiographic features of keratoameloblastoma are reviewed, along with treatment approaches and follow-up considerations. Although only a few cases have been documented, the tumor’s aggressive biological behavior and the high recurrence rate suggest that a more aggressive therapeutic approach is warranted. Patients should be informed of the importance of clinical monitoring. Surgical resection with adequate safety margins and histopathological evaluation of the margins is strongly recommended.

** Key words:**Odontogenic tumors, keratoameloblastoma, ameloblastoma, review.

## Introduction

An unusual histologic variant of ameloblastoma characterized by ameloblastic islands filled with keratin and exhibiting varying degrees of keratinization was first described by Pindborg [1] in 1970 as papilliferous keratoameloblastoma (PKAB), and later named keratoameloblastoma (KAB) by Altini *et al*. [2] in 1976. Despite the similarity of nomenclature, KAB and PKAB are morphologically distinct entities. PKAB is characterized by cystic spaces filled with necrotic debris and lined by papillary keratinized projections of odontogenic epithelium resembling ameloblastoma. The epithelium consists of cells resembling the stellate reticulum of the enamel organ and a basal layer of tall columnar, ameloblast-like cells exhibiting nuclear palisading and reversed polarity [3-6]. In contrast, KAB is histologically described as cystic follicles filled with parakeratin, orthokeratin, and necrotic material, and calcification, lined by stratified squamous epithelium with hyperchromatic, palisaded basal cells showing focal reverse polarity and subnuclear vacuolation. Peripheral areas resemble odontogenic keratocyst [4-11].

Both PKAB and KAB typically presents as painless mandibular swellings in adult patients [1-12]. Radiographically, they may appear as unilocular or multilocular lesions, sometimes irregular and with containing calcifications [5,11,13], often associated with osteolysis and cortical bone erosion. Due to their potential for recurrence, a more aggressive therapeutic approach should be considered. The most common treatment reported has been resection [2-4,7,10,12].

We report a case of recurrent KAB with an unusual histological pattern and review previously reported cases of PKAB and KAB, with an emphasis on their radiographic and histologic features, treatment, and follow-up outcomes.

## Case Report

A 46-year-old white female was referred to the Stomatology Service of a public School of Dentistry with a chief complaint of swelling in the right infratemporal fossa. The lesion had been noted approximately one year prior to the initial clinical examination. The patient reported a history of respiratory issues (long-standing bronchitis) and previous surgery for removal of a mandibular tumor six years earlier, although she was unable to specify the exact diagnosis.

**Figure 1:**
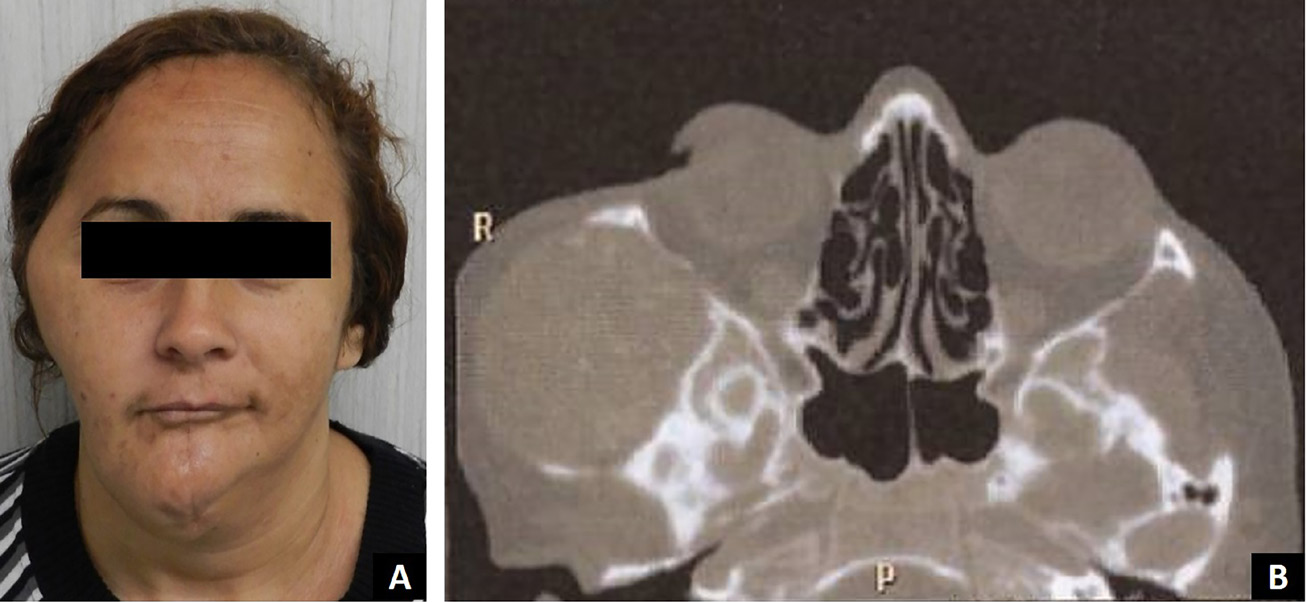
(A) Extra-oral examination: Firm well-circumscribed swelling in right infratemporal region measuring about 7 cm on its longest axis and surgical scar in the middle mandibular region. (B) Axial CT scan showing an expansile solid lesion with internal calcification in the right infratemporal fossa.

Extraoral examination revealed a firm, well-circumscribed, painless swelling in the right infratemporal region, measuring approximately 7 cm at its greatest dimension, along with a surgical scar in the mid-mandibular region (Fig. 1A). Intraoral examination showed the absence of right mandibular molars. Immediate radiographic evaluation confirmed the loss of part of the mandibular body, ramus, and right condyle.

For diagnostic purposes, histological slides from the initial surgery were retrieved. The histopathological diagnosis of the original mandibular lesion was keratoameloblastoma (KAB). Due to the rarity of this lesion, the slides were reviewed by three experienced oral pathologists, all of whom confirmed the diagnosis. A computed tomography (CT) scan was also requested, which revealed a bulky mass in the right infratemporal region with osteolysis of the zygomatic arch and areas of calcification (Fig. 1B).

**Figure 2:**
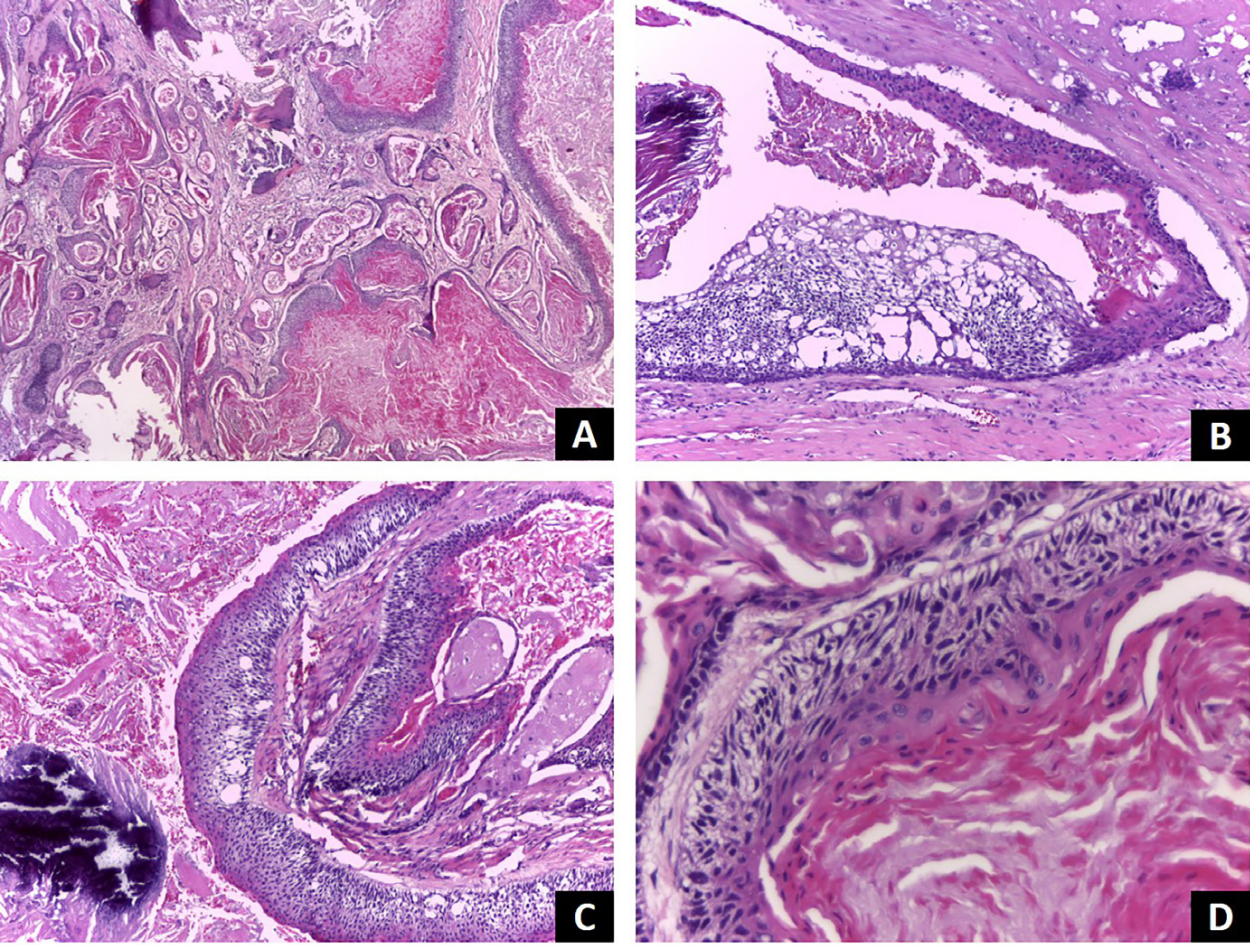
Histopatological features of Keratoameloblastoma. (A) Cystic proliferation of odontogenic epithelium; keratin and cellular debris are observed filling the cystic lumen. The lining epithelium present variable thickness and multiple cell layers. Some cystic areas are similar to ameloblastoma and other to odontogenic keratocystic (HE, 40x). (B-C) The epitheluim shows a basal cell layer with palisade colunar cells with polarized nuclei. The intermediate layer shows cells remembering stellate reticulum of enamel organ, but some areas are similar to odontogenic keratocystic. The superficial cell layer presents keratinized cells. A calcification area can be observed into the surrounding connective tissue (HE, 100x). (D) High power view showing the lining cystic epithelium organized similar to an ameloblastoma island; an intense keratinization is observed in the lumen (HE, 400x).

The patient was then referred to the maxillofacial surgery department of a public hospital, where an incisional biopsy was performed. Histopathological examination revealed a solid lesion composed of islands of columnar cells resembling ameloblasts surrounding a central area of stellate reticulum-like cells. Some islands were completely filled with keratin, and others displayed columnar to cuboidal basal cells with hyperchromatic nuclei (Fig. 2). These histological features were consistent with those observed in the previous mandibular lesion.

**Figure 3:**
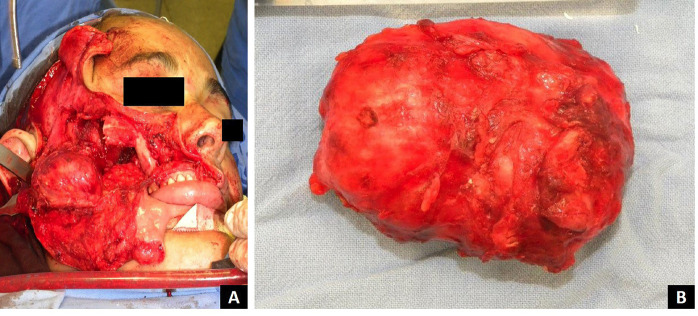
(A) Total removal of the infratemporal lesion with safety margins. (B) Surgical specimen.

**Figure 4:**
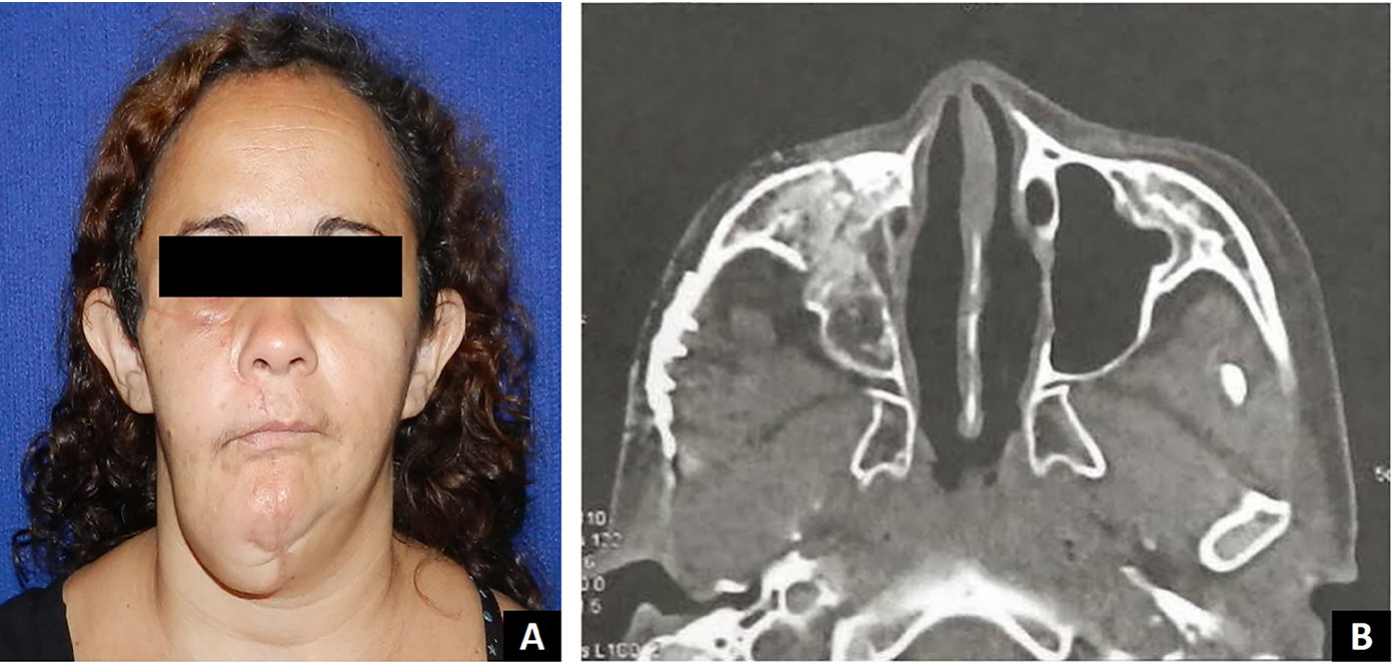
(A) Clinical follow-up of 36 months. (B) Axial CT scan of follow-up of 36 months.

A diagnosis of recurrent KAB was confirmed. Surgical treatment involved complete excision of the infratemporal lesion with safety margins, followed by reconstruction of the zygomatic arch using an autogenous calvarial (skullcap) bone graft (Fig. 3). Histopathological evaluation of the surgical specimen confirmed the same features observed in both the incisional biopsy and the original mandibular lesion. These findings supported the final diagnosis of KAB and confirmed the lesion as a recurrence of the earlier tumor. The patient has been under clinical and tomographic follow-up for 36 months, with no signs of recurrence (Fig. 4).

## Discussion

Ameloblastoma is the most common odontogenic epithelial tumor of the jaws, accounting for approximately 1% of all oral tumors and 10% of all odontogenic tumors [6,14,15]. It is generally slow-growing but locally invasive, with a high recurrence rate if not adequately treated. Its prevalence, combined with its clinical behavior, makes ameloblastoma the most significant odontogenic neoplasm [16]. Ameloblastoma is highly polymorphic due to its ability to undergo various forms of metaplasia. The stimulus for such metaplastic changes is poorly understood but has been attributed to the multipotentiality of odontogenic epithelium [12].

Understanding the clinicopathological behavior of ameloblastoma and odontogenic keratocyst may help clarify the nature of keratoameloblastoma (KAB). Some authors have expressed frustration over this entity due to the uncertainty about whether it represents an odontogenic keratocyst with ameloblastic foci, an ameloblastoma with areas resembling odontogenic keratocyst, or possibly a hybrid lesion [7,11,17]. To date, thirty-eight cases have been reported in the English-language literature under the term KAB ( Table 1). A literature search was conducted using the PubMed/MEDLINE online database for case reports and/or case series on keratoameloblastoma, published between 1970 and April 2025, without language restrictions. Among these, nine cases were identified as presenting with a variant known as papilliferous keratoameloblastoma (PKAB) [1,3-6,18-21].

This papilliferous variant was first reported in 1970 by Pindborg as an unusual type of ameloblastoma with keratinization, consisting partly of keratinizing cysts and partly of tumor islands with a papilliferous architecture [1]. Six years later, Altini *et al*. [2] described a similar lesion, but without the papilliferous component. In that case, numerous follicles of odontogenic epithelium were observed, many of which had undergone central cystic degeneration and were lined by parakeratinized stratified squamous epithelium, filled with desquamated parakeratotic cells.

Although earlier reports described lesions with histopathological similarities [22,23], the terms PKAB and KAB were first formally introduced by these two authors. An additional case of PKAB was described by Altini *et al*. [3] before the 1992 World Health Organization (WHO) classification included it among the other variants of ameloblastoma [24]. That classification clarified the distinction between KAB and the acanthomatous variant of ameloblastoma, the latter of which features extensive squamous metaplasia, sometimes with keratin formation within tumor cell islands. Although keratinization may occur in the acanthomatous type, it is not its defining characteristic. In contrast, PKAB and KAB are unique in their presentation of extensive keratinization [13,16,20].

When comparing the two KAB variants, the “KAB” type refers to a lesion with more extensive keratinization, while the “PKAB” variant is defined by the presence of microcysts lined by parakeratinized epithelium containing keratin, along with areas of non-keratinized epithelium with a papilliferous architecture.

Collini *et al*. [5] reported a recurrent case of PKAB and proposed that, due to its biological behavior, it should be reclassified as papillary ameloblastic carcinoma. This suggestion was based on features such as an increased number of mitotic Figures and extensive necrosis, which are typically considered markers of malignancy [5]. However, Gardner [25] argued that a diagnosis of ameloblastic carcinoma is justified only when there are clear dysplastic changes, which have not been observed in the reported cases of KAB.

Regarding histopathological aspects, the cases reported in the literature ( Table 1) reveal several points of particular interest. Some authors [3,5,8,11] have stated that the first three published cases lacked the typical histopathological features of ameloblastoma. Based on this view, the case reported by Norval *et al*. [4] would be considered the first to present a true PKAB, while the case by Siar *et al*. [7] would be the first to present a true KAB. Norval *et al*. [4] described their case as an unusual variant of KAB, as it showed only focal papilliferous areas and, in the authors’ opinion, may have represented an example of acanthomatous ameloblastoma.

The report by Kaku [9] lacks several important details, as it was published only as an abstract. Some reports described the presence of calcification within the lesion—an uncommon feature in ameloblastomas [5,11,13]. Additionally, root resorption, which is a typical finding in ameloblastoma, was reported in one case [6].

Our review revealed that KAB/PKAB primarily affects the mandible (71%) in young adults, with no gender predilection ( Table 1). Resection was the treatment of choice in most of the reviewed cases. Furthermore, this same treatment approach was employed in all recurrent cases, including our patient [8,13,26,27]. Only seven of the reviewed cases confirmed lesion recurrence [5,8,13,17,26-28].

It remains difficult to correlate recurrence with either the histological pattern or the treatment method used for PKAB/KAB. Among the seven recurrent cases, the papilliferous pattern was observed in only one [5]. Our presented case involved an unusual recurrence site—the right infratemporal fossa—occurring after hemimandibulectomy. A similar recurrence was reported in another case [5], in which the patient underwent two surgical resections and ultimately died from lymphoma following the detection of a third recurrence. One possible explanation for recurrence at this site is residual tumor cells in the region of the temporomandibular joint, which may have infiltrated the adjacent soft tissue.

Although KAB and its papilliferous variant are not malignant, caution is warranted when planning surgical treatment. The lesion’s biologically aggressive behavior and potential for recurrence suggest that a more aggressive approach is necessary. Patients must be informed about the importance of long-term clinical follow-up. Wide surgical resection with adequate safety margins and histopathological evaluation of the surgical margins is highly recommended.

## Figures and Tables

**Table 1:** Previously reported cases of keratoameloblastoma in literature (1970-2025).

N.	Authors	Age/ Sex	Location	Radiographic	Histopathologic	Treatment	Follow-up (months)	Recurrence
1	Pindborg JJ (1)	57/F	Right mandibular body and ramus	Multilocular	PKAB	NI	NI	NI
2	Altini M et al. (2)	28/M	Anterior maxilla	Multilocular	KAB	Resection	NI	NI
3	Altini M et al. (3)	76/M	Right mandible	Multilocular	PKAB	Resection	12	No
4	Norval EJG et al. (4)	26/F	Right mandible	Irregular	PKAB	Resection	NI	NI
5	Collini P et al. (5)	62/M	Right mandibular ramus and condyle	Irregular with calcifications	PKAB	Resection	39	Yes
6	Mohanty N et al. (6)	46/M	Right posterior mandible	Multilocular	PKAB	NI	NI	NI
7	Konda P et al. (19)	44/M	Right posterior mandible	Unilocular	PKAB	Enucleation	12	No
8	Siar CH et al. (7)	39/F	Left anterior mandible	Unilocular	KAB	Enucleation	NI	NI
9		35/F	Right maxilla	Ground glass	KAB	NI	NI	NI
10		35/M	Left mandible	Unknown	KAB	Resection	NI	NI
11		30/M	Anterior mandible	Multilocular	KAB	Resection	NI	NI
12	Said-al-Naief et al. (8)	26/M	Right posterior maxilla	Unilocular	KAB	Curettage (1st) Resection (2nd)	6	Yes
13	Kaku T et al. (9)	35/M	Right body of mandible	Unilocular	KAB	NI	NI	NI
14	Takeda Y et al. (10)	76/M	Left body of mandible	Multilocular	KAB	Resection	NI	NI
15	Whitt JC et al. (11)	45/M	Anterior maxilla	Unilocular with calcifications	KAB	Curettage	10	No
16	Adeyemi BF et al. (12)	38/M	Right posterior mandible	Multilocular	KAB	Resection	NI	NI
17	Sisto JM et al. (29)	35/F	Right posterior mandible	Multilocular	KAB	Resection	NI	NI
18	Ketabi MA et al. (14)	21/F	Right anterior mandible	Unilocular	KAB	Enucleation	12	No
19	Raj V et al. (16)	22/F	Right posterior mandible	Unilocular	KAB	Resection	24	No
20	Palaskar SJ et al. (26)	65/F	Anterior mandible	Unilocular	KAB	Enucleation (1st) Resection (2nd)	4	Yes
21	Lee C et al. (13)	56/M	Right maxilla	Irregular with calcifications	KAB	Enucleation (1st-4th) Resection (5th-7th)	40	Yes
22	Bedi RS et al. (27)	27/F	Right posterior mandible	Soft tissue tumor mass	KAB	Resection	36	Yes
23	Rathore et al. (20)	18/M	Right of mandible	Multilocular	PKAB	Local excision	24	No
24	Anajar et al. (30)	32/F	Right posterior mandible	Multilocular osteolytic	KAB	Resection	6	No
25	Parikh et al. (31)	32/F	Left palate	No evidence	KAB	Enucleation	NI	NI
26	Kuberappa et al. (21)	65/M	Right and left of mandible	Multilocular	PKAB	Enucleation	2	No
27	Robinson et al. (28)	41/F	Posterior maxilla	Unilocular	KAB	Resection	72	Yes
28		40/F	Posterior maxilla	Unilocular with calcifications	KAB	Resection	NI	No
29		45/F	Anterior and posterior of mandible	Multilocular with calcifications	KAB	Resection	NI	No
30		29/F	Anterior and posterior of maxilla	Multilocular with mixed density	KAB	Resection	NI	No
31		45/M	Anterior and posterior of mandible	Multilocular with calcifications	KAB	Only incisional biopsy	NI	NI
32		31/M	Posterior of mandible	Unilocular	KAB	Resection	NI	No
33		48/F	Posterior of mandible	Multilocular with calcifications	KAB	Resection	NI	No
34	Stojanov et al. (32)	54/M	Right maxilla	Unilocular with calcifications	KAB	Conservative excision	NI	NI
35	Moradi et al. (33)	54/F	Posterior of mandible	Unilocular	KAB	Resection with peripheral ostectomy	6	No
36	Jacob et al. (18)	42/M	Anterior and posterior of mandible	Multilocular with root resorption	PKAB	Incisional and excisional biopsy	NI	NI
37	Yamasaki et al. (17)	26/M	Right maxillary sinus to the right nasal cavity	Unilocular	KAB	Resection with peripheral ostectomy	120	Yes
38	Verma et al. (34)	14/M	Left maxilla	Multilocular	KAB	Resection	24	No
39	Netto R et al. (Present study)	46/F	Right Posterior Mandible Right Infratemporal Fossa	NI Unilocular with calcifications	KAB KAB	Resection Resection	72 36	Yes No

M: Male; F: Female; PKAB: Papilliferous keratoameloblastoma; KAB: Keratoameloblastoma; NI: Not informed.

## Data Availability

The datasets used and/or analyzed during the current study are available from the corresponding author.
